# Brachial plexus palsy after a left-side modified radical mastectomy with immediate latissimusdorsi flap reconstruction: report of a case

**DOI:** 10.1186/1477-7819-11-276

**Published:** 2013-10-15

**Authors:** Jun-Dong Wu, Wen-He Huang, Zi-Yi Huang, Ming Chen, Guo-Jun Zhang

**Affiliations:** 1 The Breast Center, Cancer Hospital of Shantou University Medical College, 7 Raoping Rd, Shantou, Guangdong 515041, China

**Keywords:** Mastectomy, Breast reconstruction, Latissimusdorsi flap, Brachial plexus injury, Complication

## Abstract

Brachial plexus injury is a rare complication during operation and anesthesia; it can occur as a result of various mechanisms such as inappropriate positioning, over-abduction and stretching the upper limbs. Brachial plexus injury can cause the poor function of the upper limb before recovery, and sometimes serious injury is unable to completely recovered the function permanently. Here, we report a female breast cancer patient who sustained a left brachial plexus palsy after modified radical mastectomy with immediate breast reconstruction with latissimusdorsi flap (LDF). The patient had fully recovered with normal function of her left upper limb six months postoperation after conservative treatment.

## Background

Iatrogenic brachial plexus injury is a rare complication during operation and anesthesia, but it causes distress and disability and often leads to litigation. The incidence of such injuries is about 9% and has increased during recent years [1]. Brachial plexus injuries have been reported in cardiac surgery, orthopedic and general surgery as well as breast reconstruction [2]. Various mechanisms such as inappropriate position, over-abducting and stretching the upper limb contribute to the occurrence of the injury. But even when reasonable care is taken to make sure patients are positioned in an appropriate position, paralysis of the brachial plexus sometimes occurs during the performance of an elective surgical procedure [3]. We report a female breast cancer patient who developed left brachial plexus palsy after modified radical mastectomy with immediate breast reconstruction with latissimusdorsi flap (LDF).

## Case presentation

**Figure 1:**
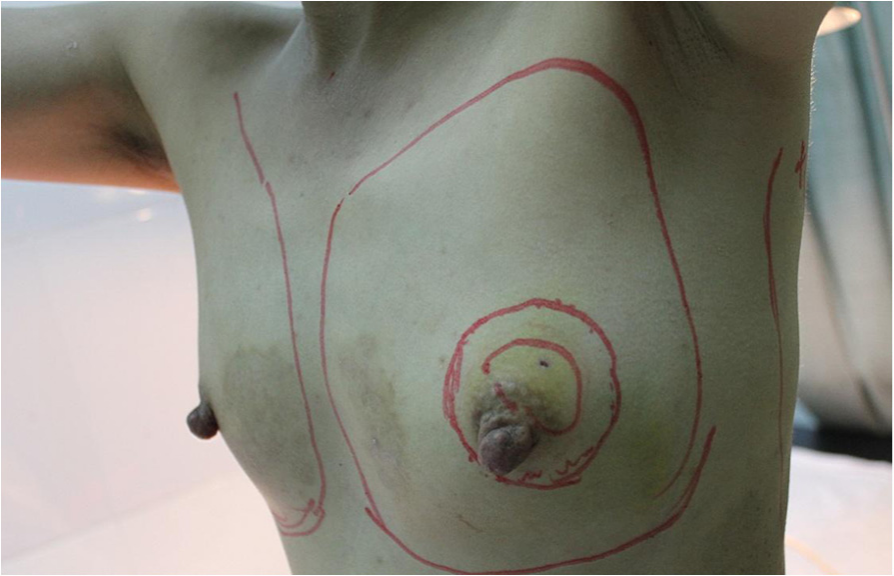
**Preoperative photograph of the patient with the breast cancer.** The tumor was located in the outer upper quadrant near the nipple (areola) region; the tumor size measured 30 mm × 28 mm in diameter (arrows); the breast size was relatively small (brassiere A cup).

A 39-year-old female patient, with a diagnosis of an invasive carcinoma of the left breast was 153 cm in height and 41 kg body weight, The tumor was located in the outer upper quadrant near the nipple (areolar) region of a relatively small breast (brassiere A cup), with the tumor size measuring 30 mm × 28 mm in diameter (Figure 1). The patient had been diagnosed with IgA-type nephropathy 15 years previously.

**Figure 2:**
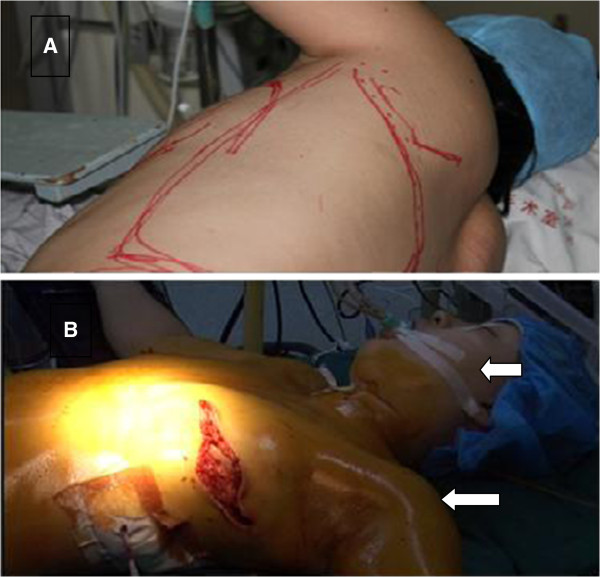
**Operative photograph of the patient in the incorrectposition.** The surgical position of the patient was changed from the right lateral decubitus position **(A)** to the original supine position **(B)**, her arm was abducted 90 degrees (arrows) but her head (arrows) was not rotated from the lateral decubitus to the supine position at this time.

The patient received a left-side modified radical mastectomy plus an immediate breast reconstruction with LDF. A routine preoperative examination showed that the patient was without evidence of musculoskeletal problems, hadno history of smoking, and had normal laboratory tests. General anesthesia was induced uneventfully with fentanyl (150 μg) and sodium thiopental (250 mg) with the patient in the supine position on the operating table. Anesthesia was maintained with isoflurane (1% to 3%)and fentanyl (total dose 300 μg) during the course of operation. The modified radical mastectomy was performed with the patient in a supine position. Both her arms were abducted and maintained at about 90 degrees, with the forearms being extended and strapped to arm boards. Her head was kept in a neutral position by a foam headrest during the modified radical mastectomy procedure. After the procedure, the patient’s position was changed to the right lateral decubitus position, with both of her arms anteriorly abducted, keeping them at an angle less than 90 degrees. Two sandbags were placed on either side of the waist on the contralateral side to stabilize the patient. During the process of harvesting the LDF, the left arm was manipulated in order to expose the operating field clearly. After harvesting the LDF, the patient was returned to the original supine position from the lateral decubitus position without head rotation (Figure 2). The flap was rotated to the chest wall and breast molding and reconstruction with the LDF were carried out. The surgery lasted for fourand a half hours, and the entire procedure was performed smoothly, without any surgical injuries to the brachial plexus during axillary dissection. The estimated blood loss was 200 ml and the patient received 2,500 ml of crystalloid and 500 ml of colloid fluid replacement and produced 1,400 ml urine.

During the following day, the patient complained of weakness of the left upper extremity and slight numbness of the fingers. She could not move the upper arm and forearm against gravity. She had weakness of shoulder abduction, wrist flexion and extension. She also had lost of sensation over the thumb and index finger and had reduction sensation in her left forearm. On the right side, the lower extremity showed normal motor and sensory function. Neurological examination of the left upper limb on admission is summarized in Table 1.

**Table 1:** Summary of neurological findings of the left upper limb

**Anatomical location**	**Muscle strength (grade)**	
Motor function	First day postoperation	Following rehabilitation
Shoulder abduction(n. axillaris)	1	4
Elbow flexion(n. musculocutaneous)	2	5
Elbow extension(n. radialis)	4	5
Wrist flexion(n. medianus)	3	5
Wrist extension(n. radialis)	2	5
Finger flexion(n. ulnaris)	4	5
Finger extension(n.radialis)	4	5
Sensory function		
Pain and light touch	Reduced	Normal
Reflexes		
Deep tendon reflexes	Reduced	Normal
Tone	Reduced	Normal

n: nevus.

**Figure 3:**
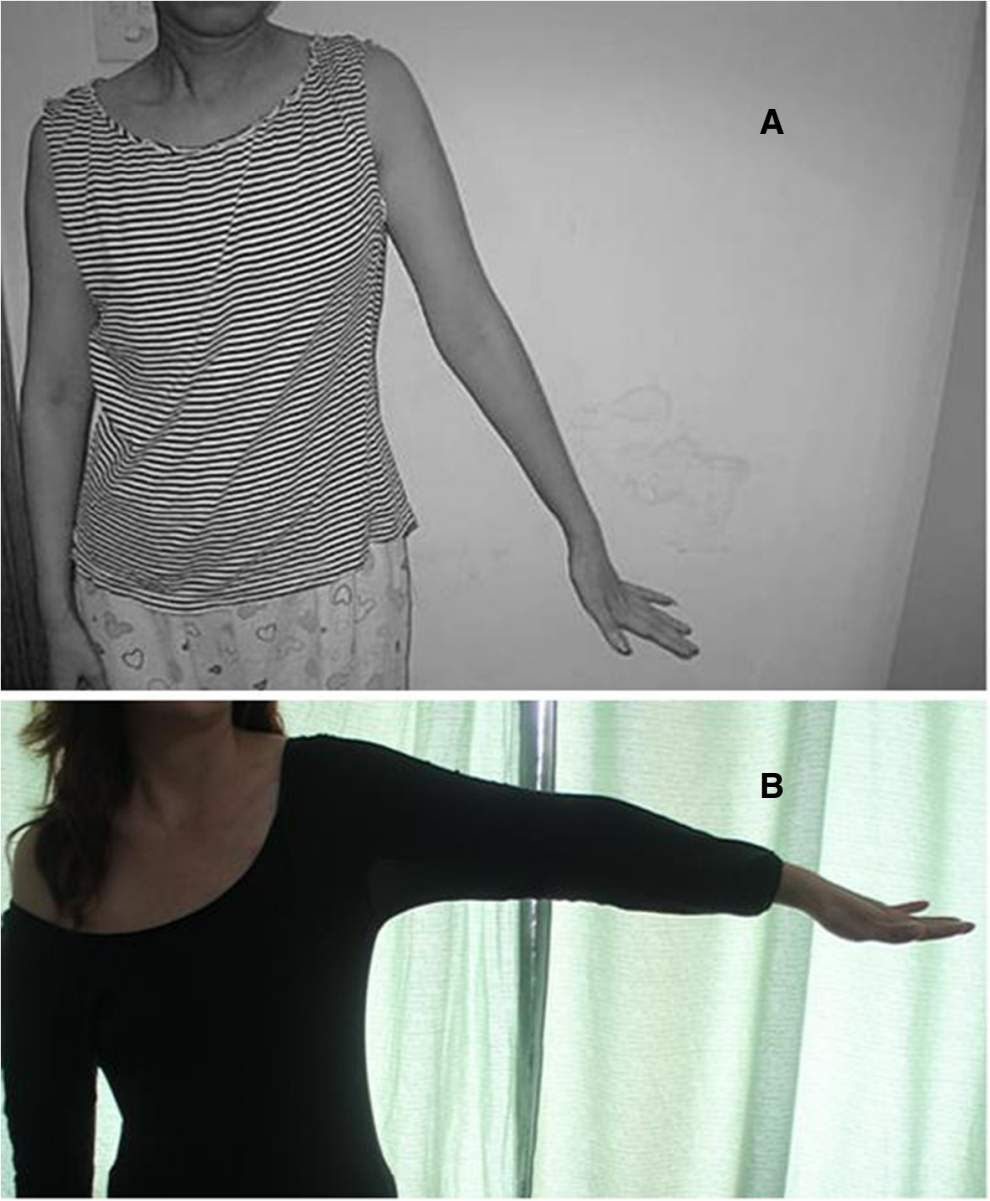
**Postoperative photograph of the patient with recovery of the left upper limb.** Three months after the surgery, the patient still had weakness of shoulder abduction (only 30 degrees abduction) but flexion and extension of elbow, wrist and fingers were normal **(A)**. Six months after the surgery, the patient’s shoulder abduction was 90 degrees, and she fully recovered normal function of her left upper limb **(B)**.

Based on the clinical symptoms, examination, axon reflex testing and electrophysiologic studies, the patient was diagnosed with a left brachial plexus injury which was located at the superior trunk. Dexamethasone (4 mg daily), vitamin B12 and mouse nerve growth factor were administrated intravenously. On the 15th day after treatment, electromyography was performed and showed no spontaneous potential in the deltoid muscle. The conduction velocity was normal for all five nerves of the brachial plexus. A physical therapy program was instituted in hospital and the patient regained normal function of the left hand six months after the surgery (Figure 3).

## Discussion

Injuries to the brachial plexus during mastectomy and axillary dissection are rare. The causes are usuallydirect surgical injuries to the brachial plexus, over-traction during axillary exploration or over manipulation of the upper extremity in order to improve exposure [4]. Traction injury from arm movement during combined mastectomy and latissimusdorsi breast reconstruction was once reported by Wilkinson [5], with two of the cases being temporary brachial plexus palsy. Here, we report a case of temporary brachial plexus palsy after immediate breast reconstruction with LDF.

Brachial plexus injury as a surgical complication was first reported by Budingerin 1894 who attributed it to the toxic effect of chloroform [6]. In 1899, the English neurosurgeon Victor Horseley reported that brachial plexus injury might be caused by stretching and/or compression of the plexus [7]. The toxicity mechanism was later abandoned while the stretch or compression mechanism was widely speculated, basing these mechanisms on three principal anatomical features. First, its superficial location makes it susceptible to direct damage by surgery. Second, the nerve roots of the brachial plexus are fixed at both their proximal sites of origin (the intervertebral foramina) and the investing fascia, muscles, and other tissues to which they are tethered distally. As a result, force applied between these points increases the likelihood of producing a stretch neuropathy. Third, the space between the first rib and the clavicle is narrow. Thus, fracture and/or displacement of the first rib can directly damage the brachial plexus [8].

Previous studies,which have attempted to investigate the relationship between arm positioning and brachial plexus neuropathy, failed to show consistent results. Jackson and Keats evaluated the stress caused by various positions on the brachial plexus in 15 cadavers. The study showed that hands-up positioning (defined as abduction of the arm to no more than 90 degrees, anterior flexion of the elbows, and elevation of the elbows six inches above the table) was associated with less tension and compression to the brachial plexus [9]. A prospective study from Kwaan *et al*. [8] was about the effect of arm position (arms following abductions to more than 90 degrees and extension) on the tension of brachial plexus. It showed that brachial plexus tension or stretching increased by progressive forearm abduction in nine fresh cadavers. In the present case, the mechanism leading to the brachial plexus injury was mostly probablya stretchingof the nerves. Both arms were in the abduction position with extension and maintained at 90 degrees abduction during the operation which lasted for fourand a half hours. The plexus was under maximum tension in this circumstance. In this procedure the left arm was manipulated to improve exposure of the operating field. Additionally, the patient was returned to the original supine position andher head was not rotated from the lateral decubitus to the supine position at this time. Studies by Kwaan*et al*. [10] showed that abduction of the arm was associated with head rotation to the contralateral side which would increase tension on the nerve.

Duration of surgical procedure could also be a risk factor of brachial palsy injury. Upton and McComas reported a double crush syndrome in 1973 [11]. A preexisting subclinical neuropathy may explain the postoperative clinically overt palsy. The periphery of the axons is continuously supplied by axonal flow of nutrients from the cell bodies in the dorsal root ganglion. Any injury that interferes with this nutrient supply can cause damage to the nerve. Such an injury may result from mechanical (nerve entrapment), metabolic (diabetes), or ischemic mechanisms. The injury may be subtle and clinically unrecognized. However, when this nerve is exposed to a second trivial injury like prolonging the operation time or continuous stretching the upper extremity, the combination of these factors may lead to significant nerve damage and clinically overt symptomatology. On the other hand, anesthesia can lead to the unconscious patient sustaining an early nerve pressure palsy which is the most common positioning injury. In our case, general anesthesia was conducted and the surgical procedure lasted for four and ahalf hours, and both arms remained in the abduction position forthe duration of the operation. These risk factors mentioned above could lead to injury to the arms in the hyper-abducted position caused by compression of the brachial plexus on both sides.

Body weight is also linked to the tension of the brachial plexus when in the respective positions of abduction, extension, or the combination of both [10], and a thinnerperson is more susceptible to brachial plexus neuropathy for nerve tension appears to be less in the heavier individuals [10]. In our case, the patient was fairly thin and weighed 41 kg which may be a contributing cause of her brachial plexus nerve injurydespite reasonable care having been taken while appropriately positioning her. In some certain conditions brachial plexus nerve injury may occur in spite of conventionally accepted positioning and padding [12].

Diagnosis of brachial plexus nerve injuries is based on clinical examination, myelography, axon reflex testing, and electrophysiologic studies. Electrophysiologic studies can detect changes in nerve function during the perioperative period, but large, prospective trials demonstrating the importance of electrophysiologic studies in the early diagnosis and prevention of brachial plexus neuropathy are lacking [13]. In our patient, the disability of abduction of the left upper extremity was obvious in the first few weeks, but electromyography (EMG) and axillary nerve conduction velocity (NCV) revealed no abnormal findings 15 days later. Symptoms during abduction of the left upper extremity persisted for six months but had completely remitted after conservative treatment.

The overall prognosis of brachial plexus neuropathies is generally good. However, prolonged recovery (up to one year) with residual symptoms sometimes occurs [14]. Hanson *et al*. [15] studied 531 cases patients prospectively and the clinical diagnosis of brachial plexus neuropathy was made in 5% (26 of 531) patients. Similarly only 1% (6 of 531) of the patients had persistent symptoms for more than four months. Vahl *et a*l. [16], in a prospective study of 1,000 patients, showed that 0.8% (8 of 1,000) patients had symptoms continuing for more than three months. Our patient’s symptoms lasted for more than four months. Six months after discharge, the patient had recovered almost full function of her left arm (summarized in Table 1).

The LDF is one of the most commonly used flap procedures and is believed to result in minimal donor-side morbidity for breast reconstruction after mastectomy [5]. It appears that, despite optimal surgical and anesthetic techniques, brachial plexus neuropathies cannot be avoided [13]. Factors that may reduce the frequency of brachial plexus neuropathies include (1) arm abduction should be limited to less than 90 degrees in supine patients and the hand and forearm kept in full supination; (2) abduction of the arm associated with head rotation to the contralateral side should be avoided; (3) padded arm boards may decrease the risk of neuropathy; (4) postoperative neurologic assessment should be performed in every patient to allow early detection and therapy of nerve lesions [3,13,17].

## Conclusions

Although most postoperative neuropathies of the upper extremities are caused by extreme abnormal positioning of the arm during intraoperative manipulation, nerve injury can still occur without this and the patient is not free of risk for neural injury, even without axillary retraction or hyperabduction of the arm. More studies are needed to specifically define risk-related maneuvers for these and other anesthetized patients [4].

## Consent

Written informed consent was obtained from the patient for publication of this Case report and any accompanying images. A copy of the written consent is available for review by the Editor-in-Chief of this journal.

## Abbreviations

LDF: Latissimusdorsi flap; EMG: Electromyography; NCV: Nerve conduction velocity.

## Competing interest

The authors declare that they have no competing interest.

## Authors’ contributions

WJD performed the operation, collected data and contributed to drafting the manuscript. WHH and ZYH participated in the operation and helped to collect data. MC was responsible for the follow-up of the patient and data preparation, literature search and participated in the preparation of the manuscript. GJZ conceived of the study, and participated in its design and coordination and helped to draft the manuscript. All authors read and approved the final manuscript.
